# COVID-19 Epidemic: Early Shift in the Socioeconomic Profile of the Affected Population

**DOI:** 10.3390/ijerph18063185

**Published:** 2021-03-19

**Authors:** Myriam Khlat, Sophie Le Coeur

**Affiliations:** Institut National d’Etudes Démographiques (Ined), 9 Cours des Humanités, 93300 Aubervilliers, France; lecoeur@ined.fr

The COVID-19 pandemic has given rise to a wealth of literature in the public health field. On theoretical grounds, it has been argued that it was exposing and amplifying social inequalities in health [1] and that it could be considered as a “syndemic pandemic” [2], which “interacts with and exacerbates existing social inequalities in chronic disease and the social determinants of health”. We would like to complete the picture by stepping back to the early stages of the epidemic, pinpointing a feature somewhat overlooked in the literature. Briefly, the idea is that the social stratification of COVID-19 cases has changed very quickly in the countries hit by the pandemic outside China, shifting from higher risks in the upper classes to higher risks in the working classes. Below are different pieces of evidence which support this vision, based on which we suggest that the theory of fundamental causes [3] is a particularly useful framework for interpreting it and drawing the relevant policy implications.

Historically, infectious diseases such as cholera, typhus, the 1918 influenza or tuberculosis have been “experienced unequally with higher rates of infection and mortality among the most disadvantaged” [2]. Recently, historians expressed their view of the COVID-19 social patterning as different from that of earlier pandemics, qualifying it as “the first pandemic that spread, to a significant extent from the affluent to the lowly” [4]. In early May, the media qualified COVID-19 as a rich man’s disease infecting the poor [4,5]. According to Joshua Loomis, author of “Epidemics: The Impact of Germs and Their Power over Humanity” [6], COVID-19 started as a rich man’s disease. Indeed, it was spread outside of China by travelers, most of whom were members of social elites and upper-middle classes [7]. In Europe, the first major cluster in late January was among a group of people gathered at a ski resort in France, who later dispersed to the United Kingdom, Spain, and other parts of France. Clusters have been reported on cruise ships, but also during various gatherings such as those organized for political campaigns preceding elections or religious events with frequent hand-shaking or hugging, affecting local political leaders or eminent personalities. Moreover, as contended by Joshua Loomis, “it did not take long to become entrenched among the poor, where most of the pandemics gravitate” [4]. Indeed, disproportionately high COVID-19 infection rates have been reported among the most disadvantaged populations in Spain, the USA and the UK [2].

The state-imposed restrictions, i.e., lockdown and social distancing, may have contributed to this spectacular shift. Indeed, “the lower people’s income, the less likely are they to be in jobs where working from home is possible” [1]. This shift may have occurred “naturally”, even without those measures, as the upper classes would have likely made use of the emerging knowledge on the risk and protective means of COVID-19, and in particular to arrange for teleworking, in contrast with lower classes, a large part of which would have been unable to work from home, particularly those in the service sector, often considered as key frontline workers. The situation of the middle classes is more mixed, as the lockdown may have been protective for those who could benefit from government-subsidized layoff, whereas those in the healthcare field have been disproportionately exposed. Nevertheless, it can be reasonably hypothesized that the state-imposed restrictions have to some extent accelerated the shift between the two most extreme social classes—the rich and the poor. In addition, the spread of the COVID-19 conspiracy theory and of non-compliance with social distancing guidelines among the least educated segments in certain populations—as observed in the USA—may have emphasized this pattern.

Quantitative data documenting the social features of the early stages of the epidemic are virtually nonexistent. In France, a significant decline in possible COVID-19 infections after the lockdown relative to before the lockdown was reported, based on a cross-sectional survey undertaken in April 2020. The decline was greater in upper classes (from 8.8% before to 4.3% after the lockdown, *p* = 0.001) than in working classes (from 6.9% to 5.5%, *p* = 0.03). As a result, the ratio in occurrence of possible COVID-19 symptoms in upper classes versus working classes reversed from 1.3 before the lockdown to 0.8 during the lockdown. In that same survey, health professionals stood out as the only group undergoing a rise over time of their percentage of possibly infected, from 6.2% before to 7.7% during the lockdown [8]. There are historical examples of similar social gradient reversal: as far as infectious diseases are concerned, this type of shift was observed for the HIV/AIDS epidemic: as soon as the modes of transmission were known, the upper classes rapidly adopted prevention measures while the epidemic was spreading in the lower classes [9]. The case of lung cancer is another example, as smoking, a highly addictive habit, has been “significantly altered by high SES groups in the name of health attainment” [10]. However, in both instances, the processes leading to reversal have taken much longer—extending over years.

COVID-19 is a new and major health hazard. Its evolving social dimension may be considered as an extraordinary illustration of the theory on the fundamental causes of disease, which theorizes that “socioeconomic disparities endure despite changing mechanisms because socioeconomic status embodies an array of resources, such as money, knowledge, prestige, power, and beneficial social connections, that protect health no matter what mechanisms are relevant at any given time” [11]. With respect to actions to be taken, the general philosophy of the fundamental cause theory is a very useful reference: “health inequalities based on socioeconomic status can be reduced by instituting health interventions that automatically benefit individuals irrespective of their own resources or behavior” [10]. Following this rationale, urgent efforts are needed to re-establish balance by ensuring that proportionately, significantly greater means are devoted to public health surveillance through screening and contact tracing, specifically tailored to the most exposed and vulnerable groups, which are currently paying the highest price, both in health and economic terms.

We would like to thank Dr. Jad Beyhum, Dr. Emilie Counil and Prof. Michel Guillot for very helpful comments on a first draft of this manuscript.
